# Herbicidal Activity of *Peumus boldus* and *Drimys winterii* Essential Oils from Chile

**DOI:** 10.3390/molecules16010403

**Published:** 2011-01-10

**Authors:** Mercedes Verdeguer, David García-Rellán, Herminio Boira, Eduardo Pérez, Sandra Gandolfo, María Amparo Blázquez

**Affiliations:** 1 Instituto Agroforestal Mediterráneo, Universidad Politécnica de Valencia, Camino de Vera s/n, C.P. 46022 Valencia, Spain; E-Mails: merversa@doctor.upv.es (M.V.); davidgr82@hotmail.com (D.G.-R.); hboira@eaf.upv.es (H.B.); 2 Centro Regional de Estudios en Alimentos Saludables, CREAS, Blanco 1623 Of.1402, Valparaíso, Chile; E-Mails: eduardoperez@creas.cl (E.P.); gandolfosandra@gmx.de (S.G.); 3 Departamento de Farmacologia, Facultat de Farmàcia, Universitat de València, Avda Vicent Andrés Estellés s/n, C.P. 46100 Burjassot, Spain

**Keywords:** essential oils, phytotoxicity, germination, seedling growth, monoterpenes

## Abstract

The essential oil composition of *Peumus boldus* and *Drimys winterii* was analyzed by means of capillary GC-FID and GC-MS. More than 96% of the total oil components (43 and 54 compounds, respectively) were identified, with ascaridole (51.17 ± 9.51), *p*-cymene (16.31 ± 2.52) and 1,8-cineole (14.45 ± 2.99) as the main compounds in *P. boldus* and γ-eudesmol (21.65 ± 0.41), followed of elemol (12.03 ± 0.34) and terpinen-4-ol (11.56 ± 1.06) in *D. winterii*. The herbicidal activity was tested against *Amaranthus hybridus* and *Portulaca oleracea*. *P. boldus* essential oil was the most phytotoxic against both weeds, inhibiting seed germination and seedling growth at all concentrations assayed (0.125–1 µL/mL). *D. winterii* essential oil did not show any effect on *A. hybridus* germination and only affected *P. oleracea* germination at the highest concentration. The results suggest the possible use of the essential oil from *P. boldus* as a natural herbicide.

## 1. Introduction

Weeds are a small group of different plants from the taxonomic point of view. They display a great adaptability to different habitats, taking advantage of the favorable conditions that occur in agricultural systems and competing with crops. Indiscriminate application of synthetic herbicides has contributed to increased resistance in weeds, also leading to gradual degradation of soil and the environment, and to hazards to human health. The secondary metabolites of plant species offer an excellent potential to develop new herbicide formulations, or as a guide towards identifying active components to obtain natural herbicides [1,2], thus numerous studies have been done with plant-derived compounds, in order to obtain synthetic herbicide substitutes for weed control [3].

*Peumus boldus* Mol. (Monimiaceae), is a native tree from the central region of Chile, being part of the sclerophyllous forests, characteristic of the Mediterranean climate. It is the source of the known crude drug Boldo folium, commonly used as a medicinal plant in Chile and reported as a herbal remedy in several pharmacopeias [4]. Infusion of boldo leaves is recommended for the treatment of gastrointestinal spasms, dyspectic and hepatobiliary disorders [5]. The main bioactive compounds of boldo leaves are flavonoids, alkaloids and essential oils. Several studies have demonstrated the antioxidant activity of flavonoids and alkaloids, particularly the aporphine alkaloid boldine [6,7,8]. The antimicrobial, fungicidal or antihelmintic effects of ascaridole, the main compound in the essential oil, is also reported [9,10], but to our knowledge, no assays of the herbicidal activity of the essential oil have been carried out so far.

*Drimys winterii* J.R.Forst. & G.Forst (Winteraceae) is a native tree of Chile that belongs to the Magallanian and Valdivian temperate rain forests. The indigenous Mapuches consider it a sacred tree with antiinflammatory, antitumoural, antibacterial and insecticidal properties. Its leaves contain flavonoids, tannins, terpenoids and also essential oil [11]. Thus, the essential oils of both native species could be promising sources of products to evaluate new activities such as the phytotoxic effect on seed germination and seedling growth of weeds. 

*Amaranthus hybridus* and *Portulaca oleracea* are two annual weeds of tropical and subtropical crops with an extensive world distribution. They have become cosmopolitan weeds distributed in a wild range of soils and climates. In Mediterranean crops these weeds show summer phenology. The aims of the present study were to determine the composition of the essential oils from two endemic plants from Chile and to compare their phytotoxic activity against *A. hybridus* L. and. *P. oleracea* L with that of plants from the Mediterranean area that we had previously tested [12]. 

## 2. Results and Discussion

The phytotoxic effects of *P. boldus* and *D. winterii* essential oils obtained from Chilean plants have been investigated against *A. hybridus* and *P. oleraceae,* two major weeds in summer crops in the Mediterranean area,. Results obtained can be explained by the very different qualitative and quantitative chemical composition of both essential oils. The essential oil composition of *P. boldus* and *D. winterii* analyzed by GC-FID and GC-MS is shown in Table 1, where the identified compounds are classified by phytochemical groups and listed in order of their elution on a methylsilicone HP-1 column.

**Table 1:** Constituents of the essential oils from *Peumus boldus*, and *Drimys winterii* by GC and GC-MS analysis.

**Compound**	**RI**	*Peumus boldus* (mean ± s.e.)	*Drimys winterii* (mean ± s.e.)
***Monoterpene hydrocarbons***		***19.71 ± 2.94***	***16.74 ± 2.43***
α-thujene	930	-	0.05 ± 0.02
α-pinene	939	0.27 ± 0.04	0.93 ± 0.23
camphene	952	0.11 ± 0.01	-
sabinene	976	1.20 ± 0.25	2.97 ± 0.68
β-pinene	979	0.18 ± 0.02	2.68 ± 0.58
myrcene	991	-	0.99 ± 0.15
α-phelladrene	1005	t	0.25 ± 0.09
δ-3-carene	1011	t	-
α-terpinene	1019	0.30 ± 0.03	1.70 ± 0.19
*p*-cymene	1027	16.31 ± 2.52	0.05 ± 0.02
limonene	1032	-	2.46 ± 0.48
β-phellandrene	1033	0.31 ± 0.13	-
*cis*-ocimene	1042	-	0.47 ± 0.05
γ-terpinene	1062	0.42 ± 0.00	3.30 ± 0.20
terpinolene	1089	-	0.91 ± 0.04
*p*-cymenene	1090	t	-
1.3.8-*p*-menthatriene	1112	0.64 ± 0.29	-
***Oxygenated monoterpenes***		***74.77 ± 4.26***	***13.95 ± 1.23***
dehydro-1,8-cineole	992	0.09 ± 0.02	-
1,8-cineole	1034	14.45 ± 2.99	0.13 ± 0.01
*cis*-sabinene hydrate	1070	0.56 ± 0.28	t
fenchone	1088	t	-
*trans-*sabinene hydrate	1097	0.51 ± 0.19	t
linalool	1100	t	0.07 ± 0.01
dehydro-sabina ketone	1121	0.83 ± 0.23	-
*cis-p*-menth-2-en-1-ol	1122	-	t
*trans*-pinocarveol	1140	1.50 ± 0.26	-
*trans-p-*menth-2-en-1-ol	1141	-	0.44 ± 0.06
camphor	1146	0.13 ± 0.02	-
sabina ketone	1160	t	-
pinocarvone	1169	0.30 ± 0.11	-
δ-terpineol	1169	0.64 ± 0.11	-
terpinen-4-ol	1179	2.15 ± 0.33	11.56 ± 1.06
thuj-3-en-10-al	1185	t	-
cryptone	1186	t	-
α-terpineol	1191	0.06 ± 0.01	1.61 ± 0.11
myrtenal	1195	0.09 ± 0.03	-
*cis*-piperitol	1196	-	t
myrtenol	1197	1.11 ± 1.33	-
*trans*- piperitol	1207	-	0.14 ± 0.01
ascaridole	1242	51.17 ± 9.51	-
*cis*-piperitone epoxide	1259	0.76 ± 0.20	-
*trans*-piperitone epoxide	1262	t	-
bornyl acetate	1288	0.08 ± 0.01	-
thymol	1293	0.06 ± 0.01	-
*p*-cymen-7-ol	1295	0.12 ± 0.02	-
carvacrol	1302	0.24 ± 0.15	-
***Sesquiterpene hydrocarbons***		***-***	***2.89 ± 0.18***
β-elemene	1392	-	0.14 ± 0.02
α-cedrene	1411	-	0.30 ± 0.04
β-caryophyllene	1419	-	0.19 ± 0.00
*trans*-β-farnesene	1459	-	0.15 ± 0.04
α-acoradiene	1465	-	t
γ-curcumene	1481	-	1.91 ± 0.14
α-curcumene	1484	-	0.17 ± 0.03
bicyclogermacrene	1496	-	0.04 ± 0.01
***Oxygenated sesquiterpenes***		***0.27 ± 0.05***	***57.82 ± 1.38***
4-*epi-cis*-dihydroagarofuran	1499	-	0.34 ± 0.02
italicene ether	1536	-	0.12 ± 0.01
elemol	1555	-	12.03 ± 0.34
*E*-nerolidol	1566	-	0.36 ± 0.01
spathulenol	1578	0.12 ± 0.03	t
rosifoliol	1602	-	0.08 ± 0.00
5-*epi*-7-*epi*-α-eudesmol	1607		t
β-oplopenone	1609	0.15 ± 0.02	-
*epi*-cedrol	1614	-	0.75 ± 0.01
10-*epi*-γ-eudesmol	1621	-	1.86 ± 0.06
γ-eudesmol	1639	-	21.65 ± 0.41
β-eudesmol	1656	-	7.27 ± 0.35
α-eudesmol	1659	-	7.35 ± 0.14
7-*epi-*α-eudesmol	1662	-	0.12 ± 0.04
β-bisabolol	1674	-	4.61 ± 0.22
α-bisabolol	1687	-	0.07 ± 0.02
drimenol	1763	-	1.18 ± 0.04
drimenin	1951	-	0.06 ± 0.01
***Aromatic compounds (C_6_-C_3_)***		***1.20 ± 0.18***	***4.83 ± 0.42***
safrole	1287	-	0.09 ± 0.01
eugenol	1360	-	1.09 ± 0.10
methyl eugenol	1406	1.20 ± 0.18	t
myristicin	1522	-	3.66 ± 0.32
elemicin	1560	-	t
***Others***		***0.34 ± 0.03***	***t***
3-octanol	993	-	t
1.4-dione-2-cyclohexene	1015	0.06 ± 0.01	-
2-nonanone	1093	0.07 ± 0.00	-
3-octanol acetate	1128	-	t
2-undecanone	1295	0.24 ± 0.03	-
**TOTAL IDENTIFIED**		**96.90 ± 1.06**	**96.24 ± 0.28**

RI: retention index relative to C_8_-C_32_*n*-alkanes on the HP-1 column. Peak area percentages were calculated in GC analysis on an apolar HP-1 column. t: trace amounts <0.03. Values are means ± standard error of three samples.

*P. boldus* essential oil is rich in monoterpene compounds, which account for more than 95% of the total oil composition. The monoterpene hydrocarbons fraction (19.71 ± 2.94) is constituted mainly by *p*-cymene (16.31 ± 2.52). Of the twelve monoterpene hydrocarbons identified, only this compound and sabinene (1.20 ± 0.25) reached percentages higher than 1%. Twenty five compounds were identified in the oxygenated monoterpenes fraction (74.77 ± 4.26). The monoterpene endoperoxide ascaridole (51.17 ± 9.51) followed by 1,8-cineole (14.45 ± 2.99), terpinen-4-ol (2.15 ± 0.33) and *trans*-pinocarveol (1.50 ± 0.26) were the main compounds. Only two oxygenated sesquiterpenes, spathulenol (0.12 ± 0.03) and β-oplopenone (0.15 ± 0.02) have been identified in *P. boldus* essential oil. This essential oil is also characterized by the absence of sesquiterpene hydrocarbons. On the other hand, only methyl eugenol, that reached a percentage around 1%, was found among the aromatic compounds. The high herbicidal activity showed by *P. boldus* essential oil correlates with the fact that a high percentage of oxygenated monoterpenes is linked to a potent phytotoxic activity [13,14,15].

Nevertheless, it is interesting to note that according to previous results [12], not only the monoterpene compounds may be responsible for germination inhibition [16,17], because previous assays with *Eucalyptus camaldulensis* essential oil, rich in the oxygenated sesquiterpene spathulenol (41.46 ± 3.94), showed that it also completely inhibited *A. hybridus* and *P. oleracea* seed germination and seedling growth [12]. 

Fifty-four compounds were identified in *D. winterii* essential oil, accounting for 96% of the total oil composition. The monoterpene hydrocarbons with 12 identified compounds almost reached 17% (16.74 ± 2.42), among them *p*-cymene only reached 0.08 in one sample, being detected in the other two as trace amounts (<0.03%). The higher percentages corresponded to γ-terpinene (3.30 ± 0.20), sabinene (2.97 ± 0.68), β-pinene (2.68 ± 0.58), limonene (2.46 ± 0.48) and α-terpinene (1.70 ± 0.19). Substantial differences on oxygenated monoterpenes were observed between the two analyzed species. This fraction (13.95 ± 1.23), represented the second most important group on *D. winterii* essential oil, with terpinen-4-ol (11.56 ± 1.06) followed of α-terpineol (1.61 ± 0.11) as the main compounds. With the exception of 1,8-cineole, *cis* and *trans*-sabinene hydrate, linalool, terpinen-4-ol and α-terpineol, which were identified in both essential oils, the oxygenated monoterpenes found in *P. boldus* were not detected in *D. winterii* essential oil. 

The greatest differences comparing both species show up in the sesquiterpene fraction. The eight sesquiterpene hydrocarbons found in *D. winterii* were absent in the three samples of *P. boldus*. With 17 identified compounds, oxygenated sesquiterpenes constituted the predominant group in *D. winterii* essential oil. This fraction (57.82 ± 1.38) contained mainly elemol (12.03 ± 0.34), β-bisabolol (4.61 ± 0.22) and eudesmol compounds: γ-eudesmol (21.65 ± 0.41), α-eudesmol (7.35 ± 0.14) and β-eudesmol (7.27 ± 0.35). 

**Figure 1:**
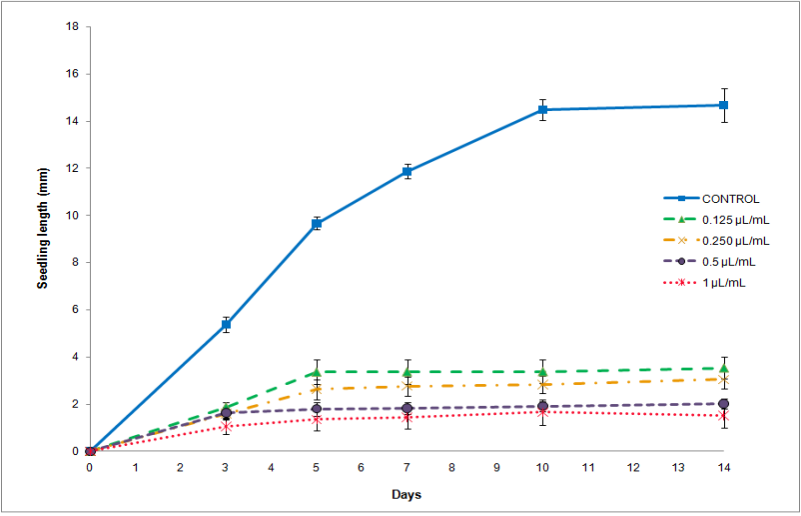
Seedling length (mm) (mean ± SE) measured for 14 days of *P. oleracea* control or treated with *D*. *winterii* essential oil at 0.125, 0.25, 0.5 and 1 µL/mL concentrations.

Finally, relative large amounts of myristicin (3.66 ± 0.32) and eugenol (1.09 ± 0.10), aromatic compounds (C_6_-C_3_) (4.83 ± 0.42) were found in *D. winterii*. From this fraction only methyl eugenol was detected in *P. boldus* essential oil. The essential oil of *D.winterii*, which also contained high percentages of oxygenated compounds was not active against *A. hybridus* germination and seedling growth. No significant differences were found between control and all concentrations applied. Nevertheless this essential oil was effective reducing *P. oleracea* germination at the higher concentrations assayed (Table 2). On the other hand *D. winterii* essential oil showed phytotoxic effects against *P. oleraceae* seedlings growth all concentrations assayed (Figure 1).

**Table 2:** Effect of *Peumus boldus and Drimys winterii* essential oils on *Portulaca oleracea* seeds germination.

Treatment	*Peumus boldus* essential oil	*Drimys winterii* essential oil
CONTROL	71.0 ± 2.9 a	71.0 ± 2.9 ab
0.125 µL/mL	0.0 ± 0.0 b	74.0 ± 5.8 a
0.250 µL/mL	0.0 ± 0.0 b	53.0 ± 6.0 b
0.5 µL/mL	0.0 ± 0.0 b	30.0 ± 6.1 c
1 µL/mL	0.0 ± 0.0 b	27.5 ± 9.9 c

Values are means ± standard error of five replicates of 20 seeds each after 14 days of incubation. Within each species, different letters indicate that means are not equal at the 95% level of probability (Fisher’s least significant difference test, LSD).

## 3. Experimental

### 3.1. Plant material

Leaves of wild *Peumus boldus* Molina and *Drimys winterii* J.R.Forst. & G.Forst. were collected in the winter of 2010 (May) at Comuna of Hualqui (36°57’36’’S; 72°55’48’’W, VIII Region, Chile) and in Quilpué (33°03’S; 71°27’W, V Region, Chile) respectively. Both areas are characterized by a temperate Mediterranean climate. 

Mature seeds of the annual weeds *Amaranthus hybridus* L. and *Portulaca oleracea* L. were collected from parent plants growing in citrus orchards of the province of Valencia (Spain), in October 2005 and August 2008, respectively. The plants were dried during 15 days at room temperature, afterwards the seeds were extracted. Uniform healthy seeds were selected and stored at 4 ºC until germination tests.

### 3.2. Oil isolation

The fresh material was subjected to hydro-distillation for three hours in a Clevenger-type apparatus, yielding (v/w) % after three distillations 1.25 ± 0.20 for *P. boldus* essential oils and 0.22 ± 0.02 for *D. winterii* essential oils. All samples were stored at 4 °C until analysis, upon which they were either diluted to 1% (v/v) in dichloromethane or their herbicidal potential was tested.

### 3.3. GC and GC-MS analyses

Gas chromatography was performed using a Perkin-Elmer Clarus 500GC apparatus equipped with a flame ionization detector (FID), a Hewlett-Packard HP-1 (cross-linked methyl silicone) capillary column (30 m long and 0.2 mm i.d., with 0.33 μm film thickness). The column temperature program was 60 °C during 5 min, with 3 °C/min increases to 180 °C, then 20 °C/min increases to 280 °C, which was maintained for 10 min. The carrier gas was helium at a flow-rate of 1 mL/min. Both the FID detector and injector port temperature were maintained at 250 and 220 °C, respectively. Gas chromatography-mass spectrometry analysis were carried out with a Varian Saturn 2000 equipped with a Varian C.S VA-5MS capillary column (30 m long and 0.25 mm i.d. with 0.25 μm film thickness). The same working conditions used for GC and split mode injection (ratio 1:25) were employed. Mass spectra were taken over the m/z 28–400 range with an ionizing voltage of 70 eV. Kovat’s retention index were calculated using co-chromatographed standard hydrocarbons. The individual compounds were identified by MS [18] and their identity was confirmed by comparison of their RIs, relative to C_8_-C_32_ *n*-alkanes, and by comparing their mass spectra and retention times with those of authentic samples or with data already available in the NIST 98 library and in the literature.

### 3.4. Biological assay

*Seed germination and growth seedling tests.* Sets of 20 seeds each with five replicates per treatment were put in Petri dishes (9 cm diameter) between two layers of filter paper (Whatman No.1) wetted with 4 mL of distilled water for germination. Essential oils of *P. boldus* or *D. winterii* were added at volumes of 0 (control), 0.5, 1, 2, and 4 μL. Based on previous assays [12], *A. hybridus* and *P. oleracea* seeds were incubated alternating 30.0 ± 0.1 °C 16 hr in light and 20.0 ± 0.1 °C 8 hr in dark. To evaluate the allelopathic potential of the essential oils, germination and seedling length data were recorded after 3, 5, 7, 10 and 14 days.

### 3.5. Statistical analyses

Tests were conducted in a randomised complete design with five replications. Data were submitted to analysis of variance (ANOVA). Percentage values were arcsin transformed. The means were compared using Fisher’s least significant difference (LSD) test (P < 0.05).

## 4. Conclusions

The present study has examined the phytotoxic effects of the essential oils of *P. boldus* and *D. winterii* two medicinal tree from Chile, against *A. hybridus* and *P. oleracea*, two serious weeds in Mediterranean summer crops. The essential oil of *P. boldus* was the most effective, completely inhibiting both *A. hybridus* and *P. oleraceae* seed germination and seedling length at all the concentrations applied (0.125–1 µL/mL). *D. winteri* showed selective activity, depending on the weed assayed. Its essential oil was not active against *A. hybridus* germination and only significantly reduced *P. oleracea* germination (57.7 and 61.3%) at the highest concentrations (0.5 and 1 µL/mL). Both species provided essential oils rich in oxygenated compounds, *P. boldus* oxygenated monoterpenes (66.39–77.61%) and *D. winterii* oxygenated sesquiterpenes (55.65–60.37%). The results corroborated previous studies suggesting that a high percentage of oxygenated monoterpenes is correlated with potent phytotoxic activity. Our *in vitro* studies suggest a possible and new alternative use of *P. boldus* essential oil in herbicidal formulations, although further experiments involving field conditions are necessary to confirm the herbicidal potential of *P. boldus.*
